# Interaction between porous silica gel microcarriers and peptides for oral administration of functional peptides

**DOI:** 10.1038/s41598-018-29345-2

**Published:** 2018-07-20

**Authors:** Kento Imai, Kazunori Shimizu, Mitsuhiro Kamimura, Hiroyuki Honda

**Affiliations:** 1 Department of Biomolecular Engineering, Graduate School of Engineering, Nagoya University, Nagoya, 464-8603 Japan; 2 Fuji Silysia Chemical Ltd., 1846, 2-Chome, Kozoji-Cho, Kasugai-Shi, Aichi 487-0013 Japan; 3 Innovative Research Center for Preventive Medical Engineering, Nagoya University, Nagoya, 464-8601 Japan

## Abstract

Functional peptides, peptides that have biological activities, have attracted attention as active ingredients of functional foods and health foods. In particular, for food applications, because orally ingested peptides are degraded by digestive enzymes in the stomach, novel oral administration methods that can prevent peptide degradation and successfully deliver them intestinally are desired. In the present study, we focused on porous silica gel, which has many useful characteristics, such as large surface area, pH responsive functional groups, size controllable pores, and approval as food additives. We investigated the possibility of using porous silica gel as a peptide degradation protective microcarrier. As a result, we found that heat treatment of the silica gel at 600 °C for 2 h remarkably enhanced the adsorbed amount of many peptides under acidic conditions, and negatively charged and highly hydrophobic peptides had suitable characteristics for oral intestinal delivery with silica gel. Finally, we demonstrated the degree of protection from pepsin degradation and found that the protection of DFELEDD peptide was 57.1 ± 3.9% when DFELEDD was mixed with the heat-treated silica gel. These results indicated that the heat-treated silica gel is promising for efficient oral intestinal delivery of hydrophobic negatively charged peptides.

## Introduction

Peptides that have biological activities are called functional peptides. Many types of functional peptides have been found to date. For example, there are those with antioxidative, antimicrobial, antihypertensive, cytomodulatory, immunomodulatory, and hypocholesterolemic activities^1–3^. They usually consist of 3 to 20 amino acid residues. These peptides are attracting attention as active ingredients of functional foods and health foods. These functional peptides are mainly obtained in protein hydrolysates. The functions of peptides in hydrolysates have been well explored and separation and purification of such functional peptides from the hydrolysates have also been actively conducted^4^.

In general, peptides are degraded by digestive enzymes, such as peptidases and proteases in the stomach when ingested orally^5,6^. Therefore, although such functional peptides are very beneficial, when taken orally, functional peptides have difficulty in reaching their target site at the luminal side of the intestinal tract or at specific peripheral organs after intestinal absorption^3,7^. Many researchers have conducted various studies to solve these problems^8,9^. One of the attractive approaches is to develop a carrier which prevents peptide degradation in the stomach. Various carriers for protection from peptide degradation, including gelatin capsules, polysaccharides, and vinyl polymers, such as carbomer, have been studied. These materials have been shown to be useful peptide carriers, especially for pharmaceutical application^10–12^. Considering the use of peptide degradation protective carriers for food application, the carriers should not change textures, flavors, or tastes of the foods. The carriers described above inevitably change food form because of contaminant solid material, undesirable high viscosity, or undesirable flavor or taste. Therefore, the development of novel carriers is desirable for peptide delivery that does not affect textures, flavors, or tastes of foods.

Porous silica gel has many useful characteristics, including a large surface area, pH-responsive functional groups, and size controllable pore size. In addition, porous silica gel is approved as a food additive. Therefore, many food industries have used this material as an anti-caking agent, for filtration in food processing, and a perfume microcarrier in food products. Recently, Schlipf *et al*. reported that porous silica gel could have a protein protective effect^13^. Green fluorescent protein (GFP), a relatively small protein, was adsorbed on various pore-sized porous silica gel, pepsin degradation was allowed, and the amount of degraded GFP was measured. The results showed that porous silica gel with approximately 10 nm pores had a peptide protective potential from pepsin by controlling pore size. In addition, various studies on the binding of silica particles and peptides have also been made^14–16^. To investigate the mechanisms of molecular recognition, especially, the events occurring at the biomolecule–inorganic interface, Puddu and Perry used three peptides identified from biopanning, and tried to identify the driving forces that govern peptide–silica binding. As a result, they elucidated the impact of binding environment (pH) on adsorption behavior of a given peptide–surface silica nanoparticle^17^.

The purpose of the present study was to investigate the possibility of porous silica gel as a peptide degradation protective microcarrier. For this purpose, we attempted to improve the affinity of peptides using silica gels with surface modification such as calcination. Furthermore, we comprehensively analyzed what properties of peptides were suitable for oral intestinal delivery using the heat-treated silica gels and synthetic peptide arrays. Peptide arrays are one of the most useful tools for analyzing various peptide-protein or peptide-material interactions^18–20^. We have used this tool to identify various material binding peptides and protein binding peptides^21–24^. In our previous paper, using the peptide array system, we investigated cellular uptake of cell penetrating peptide (CPP) conjugated tri- or penta-peptide libraries consisting of 31 peptides each with different hydrophobicity and isoelectric points (pI)^25^. As a result, we found that the uptake of the peptides by the cells varied depending on hydrophobicity and pI of the peptides. From these results, we hypothesized that we can assess peptide characteristics of peptide delivery potential by porous silica gels in the same way.

In the present study, we prepared tri-, penta-, and hepta-peptide libraries consisting of 32 peptides each with different hydrophobicity and isoelectric points (total of 96 peptides). These peptides were selected to be scattered in the whole scatter diagram. With these peptides, we explored what physicochemical characteristics of the peptides were suitable for oral intestinal delivery using the surface modified silica gels. In addition, pepsin (7.3 nm × 3.6 nm × 5.4 nm, 34.6 kDa) was applied to peptides adsorbed to silica gels (10 nm pore size) both to verify peptide protective potential of the silica gels and to quantify the amount of peptides protected from degradation. As a result, we found that the hydrophobicity and charge of the peptides and silica gels were very important characteristics to peptide delivery, and the porous silica gel had potential to protect the degradation of the peptides.

This paper shows that porous silica gel is a novel and effective tool as a peptide transport microcarrier. To the best of our knowledge, this is the first study to comprehensively investigate peptide affinity for the surface of the silica gels, by arranging the number of amino acid residues and physicochemical characteristics of the peptides.

## Results and Discussion

### Characterization of silica gels used in this study

**Figure 1:**
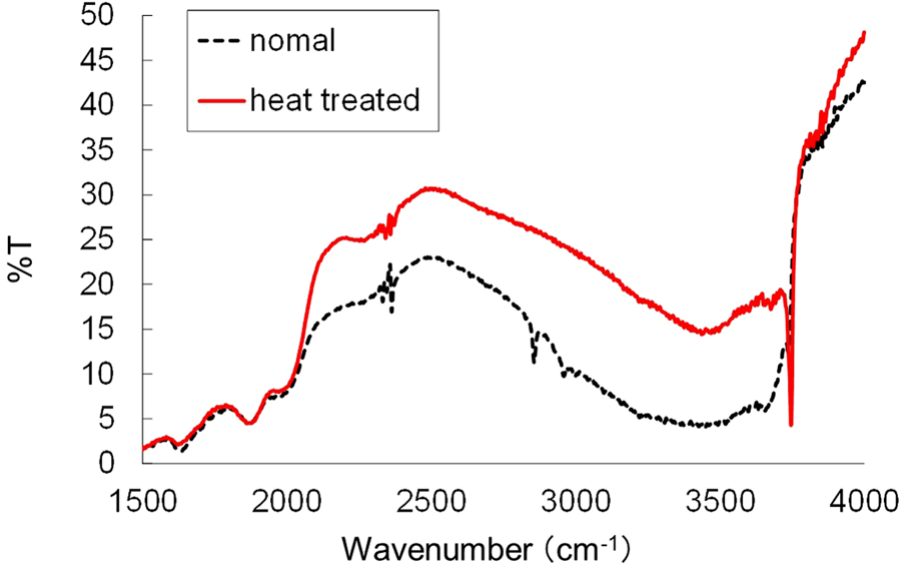
FT-IR of silica gel before and after heat treatment using diffuse reflection method. This shows the disappearance of Si-OH peaks, which are broad peaks at 3000–3750 cm^−1^.

We used two types of silica gels: normal silica gel and heat-treated silica gel that was prepared from normal silica gel by calcination at 600 °C for 2 h. The chemical composition and physical properties of normal silica gel were strictly controlled by the sol-gel method. Both silica gels had a small pore size, roughly 4 to 11 nm (mainly 10 nm) (Fig. S1). As shown in Table 1, the structural properties, including particle size, pore size, surface area, and pore volume were not different between the two particles. FT-IR spectrum shows the disappearance of Si-OH peaks, which were broad peaks at 3000–3750 cm^−1^, for heat-treated silica gels (Fig. 1). These results indicated that the heat-treated silica gel had comparable structural properties to normal silica gel, although it had a more highly hydrophobic character than did the normal one.

**Table 1:** Structural properties of silica gel.

Silica gel	Size (μm)	Pore size (nm)	Surface area (m^2^/g)	Pore volume (ml/g)
SMB-100-5	5	10.3 ± 0.2	304 ± 8.7	0.78 ± 0.02
SMB-100-5 (heat treated)	5	10.9 ± 0.4	267 ± 17.7	0.72 ± 0.02

### Tripeptide assay

**Figure 2:**
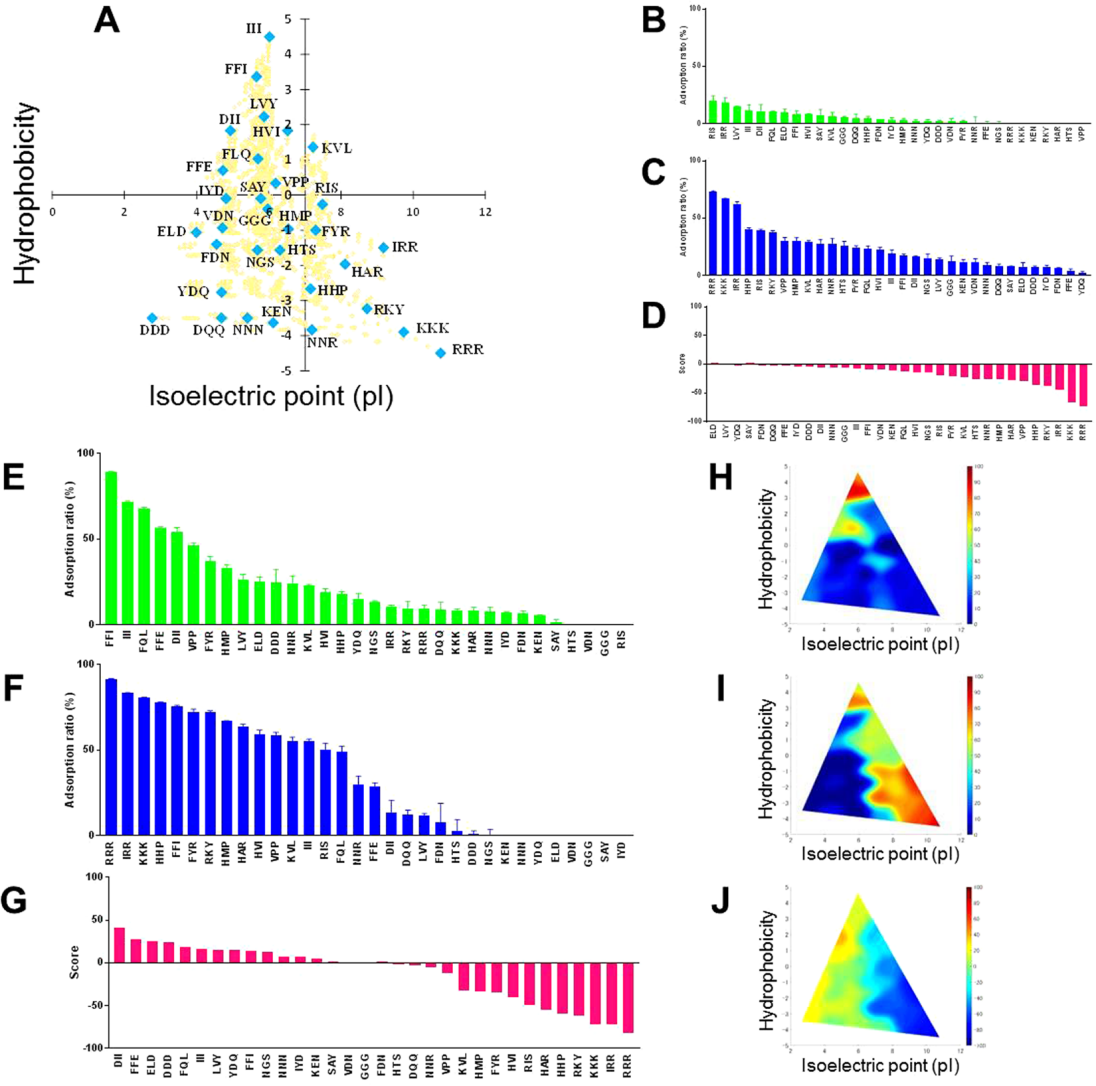
Peptide adsorption ability by adding tripeptide sequences. Green bars represent the pH 2.1 condition. Blue bars represent the pH 7.4 condition. Pink bars represent score values. Each adsorption ratio was calculated using the fluorescence value before adding the silica gel and after adding the silica gel. (**A**) A chart of all the tripeptide sequences classified by hydrophobicity versus isoelectric point (pI). Orange squares denote all tripeptides. Light blue diamonds denote representative peptides. (**B**) Peptide adsorption ability on normal silica gel (pH 2.1). (**C**) Peptide adsorption ability on normal silica gel (pH 7.4). (**D**) Score values for normal silica gel. (**E**) Peptide adsorption ability on heat-treated silica gel (pH 2.1). (**F**) Peptide adsorption ability on heat-treated silica gel (pH 7.4). (**G**) Score values for heat-treated silica gel. (**H**) Color map based on the results of (**E**). (**I**) Color map based on the results of (**F**). (**J**) Color map based on the results of (**G**).

We firstly investigated the adsorption ratio of the 32 tripeptides with various hydrophobicity and isoelectric points (Fig. 2A and Table S1) in normal silica gel at pH 2.1 and 7.4. Then, to estimate the efficiency for oral intestinal delivery, we determined the score value by subtracting the adsorption ratio of pH 7.4 from that of pH 2.1 (See materials and methods) (Fig. 2B–D). All score values of the tripeptides were less than 3.0% (Fig. 2D). These results indicated that normal silica gel was unsuitable for efficient oral intestinal delivery of peptides.

As shown in Fig. 2B, the adsorption ratio at pH 2.1 was considerably low; the highest adsorption ratio was only 19.8 ± 4.4% for RIS and the adsorption ratios of 26 tripeptides were less than 10.0%. This was the primary cause of the low score value. The hydrophobic interactions are significant at the silica-peptide interface^17^. 26 tripeptides showing less than 10% of adsorption ratio at pH 2.1 were relatively hydrophilic. These average hydrophobicity was −1.48 and that of other 6 tripeptides was 1.31. On the other hand, relationship between pI was weak. The pI average of 26 tripeptides and 6 tripeptides was 6.21 and 6.53, respectively. Therefore, we next employed the heat-treated silica gel that had a higher hydrophobic surface (Fig. 1) and determined the score value (Fig. 2E–G). We found that the score value was dramatically improved using the heat-treated silica gel; the top fifth of the score values were 40.9 for DII, 27.6 for FFE, 24.9 for ELD, 23.8 for DDD, and 18.7 for FQL. As shown in Fig. 2E, the adsorption ratio at pH 2.1 was high using the heat-treated silica gel, as we expected.

Although the number of tripeptides that had positive score values was increased using the heat-treated silica gel, almost half of the tested tripeptides had negative score values (Fig. 2G). We assumed that the score values varied depending on the physicochemical properties of the tripeptides. Color maps in Fig. 2H–J show the effects of the physicochemical properties of tripeptides on the adsorption ratio at pH 2.1, 7.4 and score value. Under the pH 2.1 condition, the upper part of the color map had a high value, indicating that hydrophobic interaction was a dominant factor (Fig. 2H). Under the pH 7.4 condition, the lower left part of the color map shows a low value, indicating that anionic and hydrophilic tripeptides, peptides with lower pI and lower hydrophobicity, were difficult to bind to the surface of heat-treated silica gel (Fig. 2I). From these results, the upper left had a high and the lower right had a low score value on the color map (Fig. 2J). These results indicated that the tripeptides with high hydrophobicity and anionic charge were suitable for oral intestinal delivery using the heat-treated silica gel.

Rimola *et al*. reported a review paper on the effect of silica surface features on the adsorption of biomolecules, such as polypeptides^16^. In pH 7, they mentioned that peptides with higher pI values (i.e. those that contained Lys and Arg residues) were attracted more strongly for those surfaces that exhibited larger surface density of negatively charged Si-O^−^ groups. This tendency was also obtained in our experiments as described in Fig. 2C. When surface hydrophobicity of silica gel was increased by heat treatment, the adsorption ratio of some peptides significantly increased at pH 7. All 5 tripeptides including F were raised in the ranking, while only 1 peptide among 13 including L, I and V was remarkably raised. This peptide was FFI. Hydrophobicity of F is 2.8 and not so high compared with L(3.8), I(4.5) and V(4.2). This means that the contribution of electrostatic interactions to peptide adsorption was not varied by heat treatment of silica gel, and the hydrophobic interaction reinforced the adsorption of peptide including F. This is supported that the adsorption of Phe was mainly driven by hydrophobic interactions between the non-polar side chain and the siloxane Si-O-Si surface groups as described by Rimola *et al*., since siloxane group was increased by heat treatment.

### Long residue peptide assay

**Figure 3:**
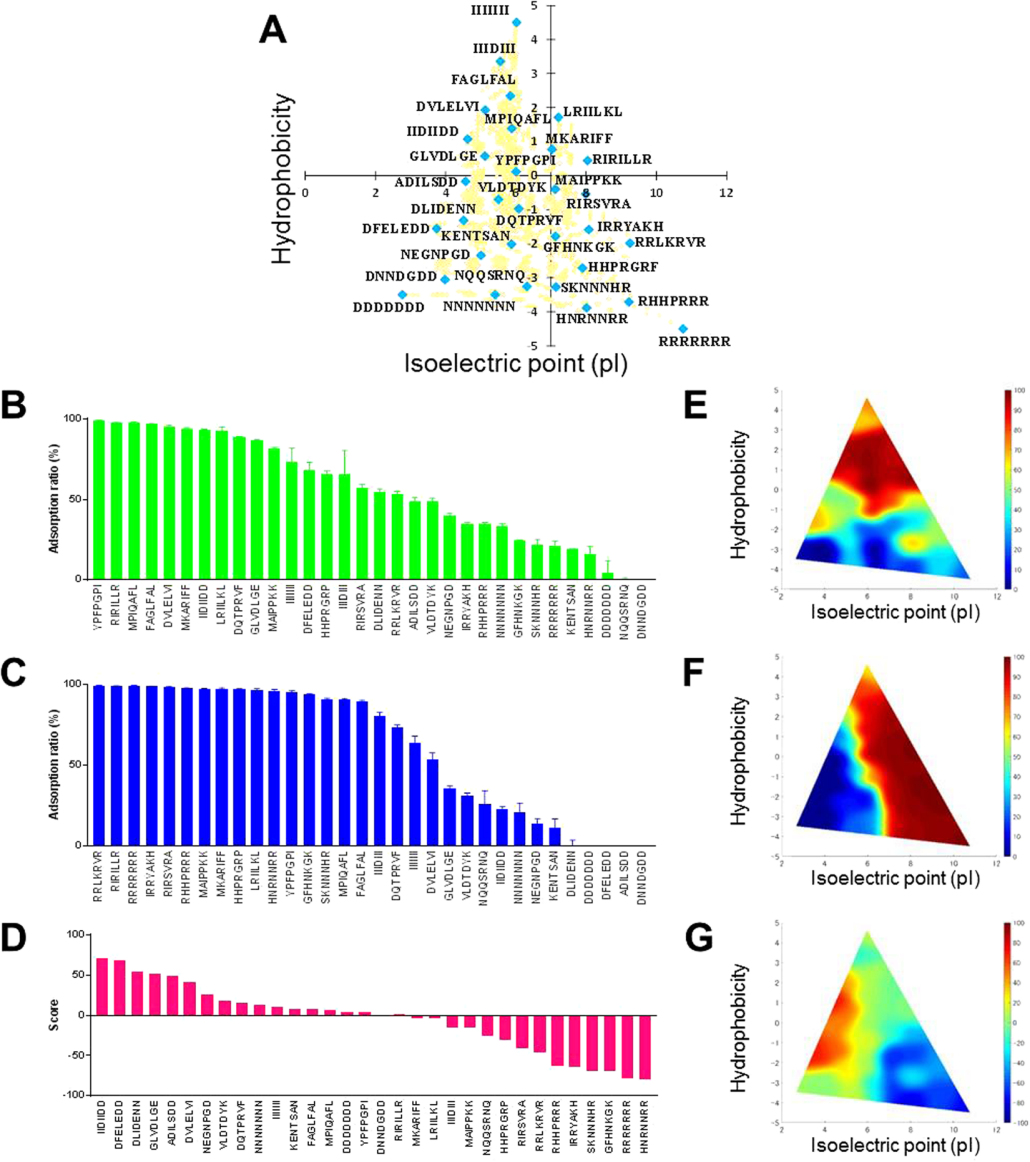
Peptide adsorption ability by adding heptapeptide sequences. (**A**) A chart of the heptapeptide sequences classified by hydrophobicity versus isoelectric point (pI). Orange squares denote all tripeptides. Light blue diamonds denote representative peptides. (**B**) Peptide adsorption ability on heat-treated silica gel (pH 2.1). (**C**) Peptide adsorption ability on heat-treated silica gel (pH 7.4). (**D**) Score values for heat-treated silica gel. (**E**) Color map based on the results of (**B**). (**F**) Color map based on the results of (**C**). (**G**) Color map based on the results of (**D**).

We next investigated whether the number of residues of the peptides affected the physicochemical properties of the peptides suited for oral intestinal delivery using heat-treated silica gel. We prepared 32 pentapeptides (Fig. S2A and Table S2) and 32 heptapeptides (Fig. 3A and Table S3) with various hydrophobicity and isoelectric points. As a result, for both pentapeptides (Fig. S2B–G) and heptapeptides (Fig. 3B–G), the peptides that had both hydrophobic and negatively charged properties had high score values, whereas the peptides that had positively charged properties had low score values. These tendencies were closely similar to that of tripeptides (Fig. 2J). However, the magnitude of the score value increased as the length increased (Figs 2G, S2D and 3D). For example, the average score values of the top five sequences were 27.2% for tripeptides, 46.5% for pentapeptides, and 58.6% for heptapeptides. This was caused by the increase in the amount of peptides adsorbing to the surface of heat-treated silica gel at pH 2.1 (Figs 2E, S2B and 3B). This may have occurred because of the increase in the number of amino acids, which basically caused greater peptide hydrophobicity.

### Adsorption isotherms

**Figure 4:**
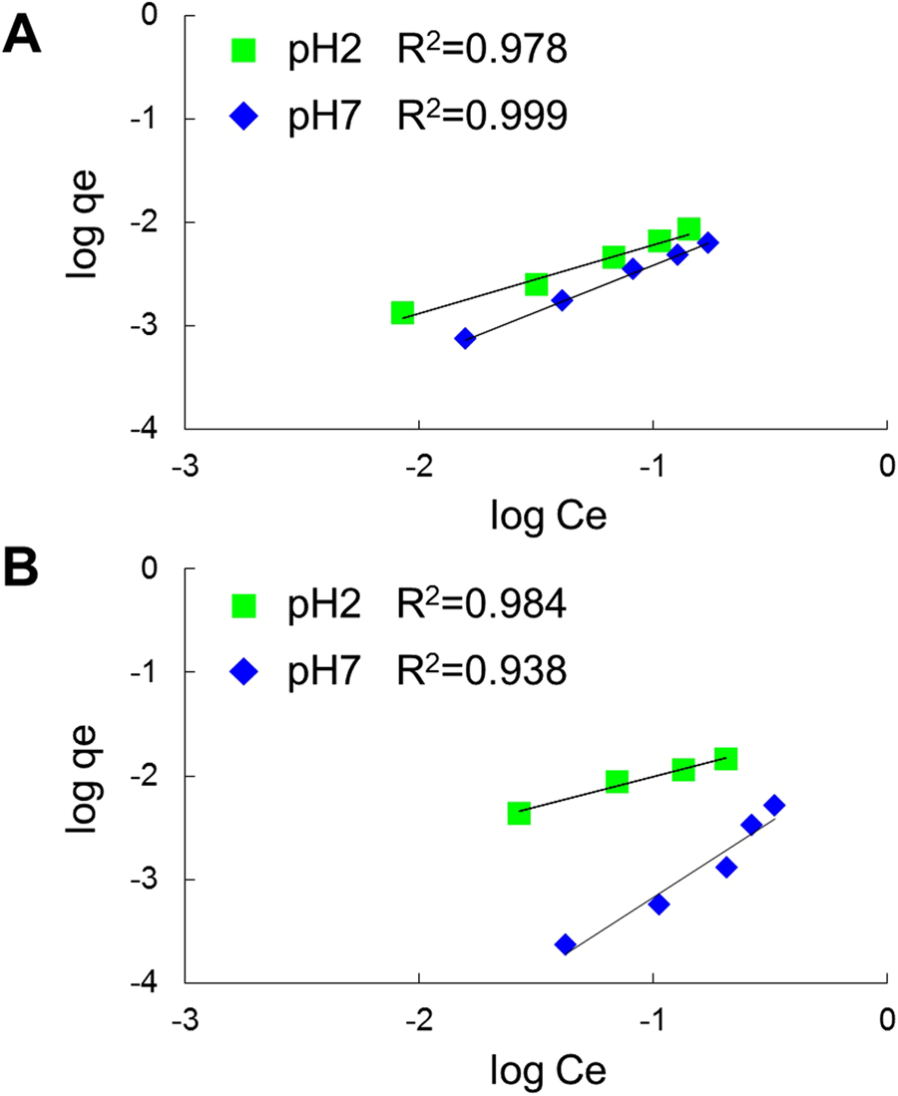
Freundlich fitting of isotherms for peptides. (**A**) DII (representative of tripeptide), (**B**) DFELEDD (representative of heptapeptide).

We next determined the adsorption isotherms of the peptides on the surface of the heat-treated silica gel at pH 2.1 and 7.4. In these experiments, DII and DFELEDD, which had high score values (Figs 2G and 3D), were used. Adsorption isotherms were plotted according to the Freundlich equation for DII (Fig. 4A) and DFELEDD (Fig. 4B). In addition, we also determined the adsorption isotherms of VLDTDYK and HNRNNRR, which have completely different physiochemical characteristics from DFELEDD, and found that the data fit the Freundlich model (R^2^ > 0.95) (Fig. S3).

Freundlich equation is an empirical equation, which indicates heterogeneity of binding sites^26^. We considered the driving force of peptide adsorption on silica gel was both of hydrophobic interaction and charge from the results described in Figs 2, 3 and S2. Therefore our consideration which peptide adsorption was ruled by the heterogeneity of binding site was strongly supported by the results that adsorption isotherm was fitted by Freundlich equation. In another previous study, Daifullah *et al*. mentioned that a multi-layer adsorption was indicated by Freundlich equation since the Freundlich equation could not achieve a plateau^27^. Therefore it is considerable that peptides adsorbed to the heat-treated silica gel could be bound by multilayer adsorption.

### Evaluation of adsorption and desorption of peptide

In the experiments above, the score value obtained by subtracting the adsorption ratio of pH 7.4 from that of pH 2.1 was used as an index to evaluate the ability of silica gel for oral intestinal peptide delivery. Next, to investigate whether the score value represented the ability of peptide release from the silica gel, actual release experiments of peptides adsorbed on heat-treated silica gel at pH 2.1 were performed at pH 7.4. The peptides were adsorbed to the heat-treated silica gel under pH 2.1 condition, then the silica gel was suspended in the pH 2.1 buffer and the pH 7.4 buffer continuously, and the amount of released peptides from the silica gel was quantified. We used three heptapeptides with different score values for this analysis (Table 2). Among these peptides, VLDTDYK has been reported as a functional peptide with angiotensin I-converting enzyme (ACE) inhibitory activity^28^.

**Table 2:** The correlation between “Score” and the amount of “Release peptide”.

Sequence	Score	Release peptide (mg/g)
DFELEDD	68.0	1.35 ± 0.04
VLDTDYK	17.8	0.34 ± 0.16
HNRNNRR	−80.4	0.10 ± 0.08

As a result, the heat-treated silica gel released 1.35 ± 0.04 mg/g of DFELEDD, 0.34 ± 0.16 mg/g of VLDTDFYK, and 0.10 ± 0.08 mg/g of HNRNNRR at pH 7.4 (Table 2). This value was the expected amount of peptide ultimately released in the intestine. Because the peptide with the higher score value released much more, it was suggested that the score value represented the ability of peptide release from the silica gel. Considering these results, color maps for score values (Figs 2J, S2G and 3G) would be useful not only for estimating the peptide sequences that are appropriate for oral intestinal peptide delivery, but also for predicting the amount of the functional peptides released from the silica gel.

Puddu and Perry elucidated the impact of binding environment (pH) on adsorption behavior of a given peptide–surface silica nanoparticle, and the prevailing interactions (i.e., electrostatic or hydrophobic/hydrogen bonding) and their relative contribution to the binding event are governed by the identity of the peptide itself, the substrate’s surface functionality (hydrophilic or hydrophobic), and the peptide bulk concentration and solution bulk pH with three peptides identified from biopanning^17^. In addition, they also reveal that intrinsic bias toward positively charged sequences in the elution conditions used in the biopanning protocol.

This study strongly supports our results that the hydrophobicity of silica gels surface and bulk pH have an effect on peptide adsorption. In this study, we evaluated peptide adsorption using 96 peptides with different hydrophobicity and isoelectric points (Figs 2J, S2G and 3G). These results are high value in evaluating the interaction between peptide and silica gel.

### Peptide protective effect of porous silica particle

**Figure 5:**
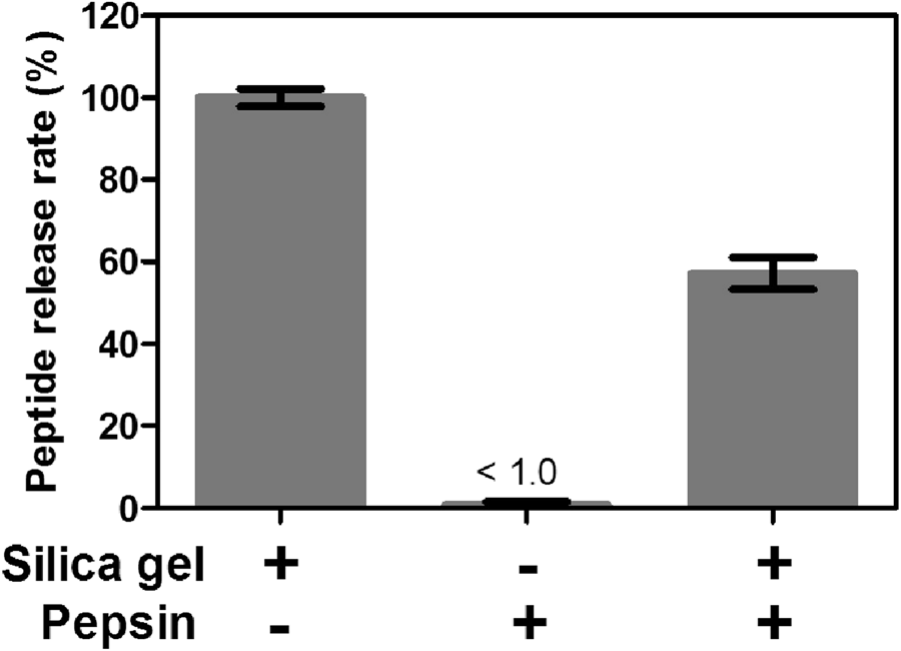
The amount of peptides released from silica gel after degradation. Scenario with no addition of pepsin was estimated as 100%.

Finally, we investigated whether the heat-treated silica gel used in this study protected the peptides that were adsorbed to them from pepsin degradation. We used DFELEDD with a high score value (Fig. 3D). The peptide was adsorbed to the heat-treated silica gel, incubated for 60 min in simulated gastric fluid containing pepsin, and then transferred to the pH 7.4 buffer that mimics the intestinal environment. As shown in Fig. 5, most of the DFELEDD was degraded without the silica gel and the peptide did not remain under the intestinal environment (<1.0%). In contrast, the degradation of the peptide was inhibited under the condition with silica gel and 57.1 ± 3.9% of the peptide was released. This occurred because the peptide was adsorbed to the pore surface of the heat-treated silica gel. Schlipf *et al*. reported EGFP (2.4 nm diameter × 4.2 nm) protection from proteolytic attack by Pepsin A (7.3 nm × 3.6 nm × 5.4 nm) by the mesoporous silica with 7.3 nm pores^13^. They discussed that larger-pored materials (>9 nm) provide diminishing protection for EGFP, and the protection is greatly reduced with increasing pore size and pore size distribution breadth. Therefore it is considerable that 57.1 ± 3.9% protection by silica gel with 10 nm average pore size will be increased by using silica gel with smaller pore size and narrower distribution breadth.

### Adsorption and desorption mechanisms

**Figure 6:**
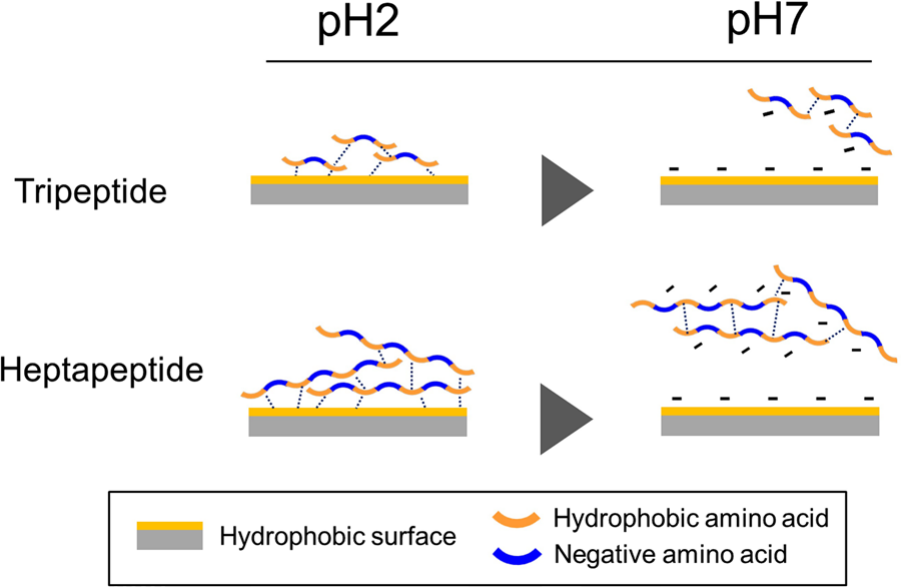
Adsorption and desorption mechanisms of hydrophobic and negative charged peptides by heat-treated silica gel.

On the basis of our experimental results, we propose an adsorption and release model for hydrophobic negatively charged peptides on the surface of silica gel (Fig. 6). The heat-treated silica gel drastically improved the adsorption of hydrophobic peptides at pH 2.1 (Fig. 2B,E). By calcining, a part of the silanol groups on the silica gel surface obtained a siloxane structure, resulting in the surface becoming more hydrophobic^29,30^. Thus, under acidic conditions, it is believed that the remaining silanol groups are not ionized, and the hydrophobic interaction between the peptides and the surface of heat-treated silica gels become more intense. Under neutral conditions, the remaining silanol groups are deprotonated and negatively charged, and thus, hydrophobic negatively charged peptides were likely to be released because of the electrical repulsion.

For oral intestinal delivery of functional peptides, there are considerably two approaches such as (1) designing a new silica gel to improve delivery characteristics including surface functionalization^31^, (2) screening functional peptides suitable for the characterization of silica gels. For food applications, chemical modification of silica gel is not an appropriate method because of the difficulty of safety assurance as food material. In our research, we examined peptide screening and investigated oral intestinal delivery of such peptides using silica gels without surface modification. Many researchers have screened wide variety of peptides, so that we could change physiological characteristics of peptides without losing its function.

## Conclusion

In this study, we investigated the possibility of porous silica gel approved as food additives as a microcarrier for oral intestinal delivery of functional peptides. We used tri-, penta-, and heptapeptides, 32 of each, with different physicochemical characteristics, including charge and hydrophobicity. We found that heat treatment of the silica gel at 600 °C for 2 h remarkably enhanced the adsorbed amount of many peptides under acidic conditions and negatively charged and highly hydrophobic peptides had suitable characteristics for oral intestinal delivery with silica gel. These adsorption properties were consistent with prior work on the effect of silica surface features on the adsorption of oligopeptides^14,16^. In this study, we investigated the adsorption properties between various peptides and silica gel for each residue number. To the best of our knowledge, this is the first study to comprehensively investigate peptide affinity for the surface of the silica gels, by arranging the number of amino acid residues and physicochemical characteristics of the peptides.

Currently, silica nanoparticles were expected to be used in various fields (e.g. DDS, medical imaging, tissue engineering)^15^. In addition, various applications have also been investigated for peptides that are highly bonded to silica; for example, protein patterning on glass substrates^32,33^, peptide tag for protein purification using silica as a solid phase^34,35^, protein immobilized tag for immobilized enzyme process^36^ etc. In these studies, a detailed understanding of the interaction between silica and biomolecules is very important. For that purpose, this result can be a new knowledge to understand the interaction between peptide and silica, and will be an important guide for designing tag peptides.

## Materials and Methods

### Characterization of silica gels

Porous silica gel, SMB-100-5 was supplied by Fuji Silysia Chemical LTD., Japan. SMB-100-5 was granulated after addition of NaOH to adjust the isoelectric point to pH 9. Heat treated silica gel was created by calcining at 600 °C for 2 h. Nitrogen adsorption–desorption isotherms were measured using a surface area and porosity analyzer. The pore size distribution was calculated using the Barrett-Joyner-Halenda model, and specific surface area was calculated by the Brunauer-Emmett-Teller method. Surface chemistry was analyzed by infrared spectroscopy (FT/IR 6100; JASCO, Japan).

### Synthesis of the peptide library

Peptide arrays were synthesized by using a cellulose membrane and a spot synthesizer (Intervis, ASP222, Cologne, Germany) as previously described^37^. After punching, each of the resulting peptide-containing disks (peptide spots) was placed in a single well of a 96-well plate filter (MSRLN0410; Merck Millipore, Germany) and 180 μL of buffer solution was added (in the pH 2.1 experiments, 100 mM phosphate buffer was used, in the pH 7.4 experiments, PBS was used). After 1 h incubation at room temperature, the solution containing peptides was released from the disk and filtered into a 96-well plate by vacuum filtration. Each filtrate was used for peptide adsorption experiments.

### Creation of peptide scatter diagram and color map

To generate the comprehensive peptide scatter diagram, we chose two indices; hydrophobicity^38^ and isoelectric point^39^, which are basic indices of the various properties of amino acids (e.g., polarity, log P, molecular weight). For example, all tripeptides in the scatter diagram were plotted on the basis of the following equations:

$${\rm{Hydrophobicity}}=({{\rm{X}}}_{{\rm{i}}1}+{{\rm{X}}}_{{\rm{i}}2}+{{\rm{X}}}_{{\rm{i}}3})/3$$

$${\rm{Isoelectric}}\,{\rm{point}}\,({\rm{pI}})=({{\rm{Y}}}_{{\rm{i}}1}+{{\rm{Y}}}_{{\rm{i}}2}+{{\rm{Y}}}_{{\rm{i}}3})/3$$

where X_i1_, X_i2_, and X_i3_ were the hydrophobicity values of the 1st, 2nd, and 3rd amino acid from the N-terminal end of peptide_i_, and Y_i1_, Y_i2_, and Y_i3_ were the isoelectric point values of the same amino acid peptide_i_, respectively. Thus, X_i_ and Y_i_ indicated the average hydrophobicity and pI of the constituent amino acids of peptide_i_, respectively.

In our scheme, to prevent enzymatic degradation in the stomach and allow transport to the intestine, peptides must remain on the silica gel in the stomach environment (acidic condition). In addition, peptides also needed to be released from the silica gel in the intestinal environment (neutral condition). Therefore, we speculated that the amount of adsorption must be high in the acidic environment (pH 2.1) and low in the neutral environment (pH 7.4). The larger the difference between these two values were for the peptides, the more suitable the peptide was for intestinal delivery. To evaluate these two indicators simultaneously, we defined “Score value” as defined below.

$${\rm{Score}}\,{\rm{value}}={\rm{pH}}\,2.1\,{\rm{adsorption}}\,{\rm{amount}}-{\rm{pH}}\,7.4\,{\rm{adsorption}}\,{\rm{amount}}$$

Therefore, both peptide and silica gel were mixed together under acidic and neutral conditions, and the amount of peptide adsorbed on silica gel was quantified. After calculating the score values, we classified all 8000 tripeptides by isoelectric point versus hydrophobicity and plotted the values (orange squares). Thirty-two representative peptides (light blue diamonds) that were dispersed over the entire map area were selected. After the experiments, we created 3D color maps with MATLAB.

### Peptide adsorption experiments using peptide array

Porous silica gel was suspended in buffer solution at 100 mg/mL. The peptide solution that was released from the peptide disk as described above was utilized. 150 μL of the peptide solution and 50 μL of the suspensions (for the reference, add 50 μl of buffer solution) was mixed and shaken vigorously. The mixture was left to equilibrate for 5 min at room temperature. The incubation time of 5 min was long enough to reach equilibrium. After being centrifuged at 10000 rpm for 1 min, the amount of adsorbed peptide was determined by measuring the amount of peptide remaining in the supernatant after adsorption. The amount of peptide was quantified by fluorimetric assay^40^. Under pH 7.4 experiments, 10 μL of fluorescamine (5 mg/mL in acetone) was added to 150 μL aliquot of the supernatant in a 96-well plate, and the fluorescence intensity was measured at 355 nm in excitation and 460 nm in emission (Fluoroskan Ascent TM Microplate; Thermo Fisher Scientific). Under pH 2.1 experiments, before fluorimetric assay, the supernatant pH was adjusted to 7.4. 200 μL of 0.1 N NaOH was added to 150 μL of the supernatant. After that, 150 μL of solution was taken out and all assays were repeated three times to guarantee their repeatability, and data are presented as mean values and standard deviation (SD).

### Peptide adsorption isotherms

To investigate adsorption isotherms, PBS solution containing peptide purchased from GL Biochem Ltd., Shanghai was utilized as peptide solution. The mixture of 50 μL of silica suspensions (100 mg/mL) and 150 μL of peptide solution ranging 0.05–0.5 mM was prepared. The mixture was left to equilibrate for 5 min at room temperature. After being centrifuged at 10000 rpm for 1 min, the adsorbed amount was investigated according to the method described above. The amount of peptide adsorbed per unit mass q_e_ (μmol/mg) was determined using the following equation:

$${{\rm{q}}}_{{\rm{e}}}={\rm{V}}({{\rm{C}}}_{0}-{{\rm{C}}}_{{\rm{e}}})/{\rm{M}}$$

Here, the initial concentration, C_o_ (mM) and the equilibrium concentration, C_e_ (mM) were calculated using the calibration curve. M was the mass of adsorbent used (mg), and V was the volume of the peptide solution (mL).

### Peptide adsorption and desorption experiment

The mixture of 150 μL of silica suspension (25 mg/mL) and 150 μL of peptide solution (0.5 mM) was prepared and shaken vigorously, and left to equilibrate for 5 min at room temperature. After being centrifuged (10000 rpm 1 min) to separate the supernatant and silica gel, 300 μL of phosphate buffer (pH 2.1) was added to the silica gel and shaken vigorously, and left to equilibrate for 5 min at room temperature. This was the acidic release step. Next, the mixture was separated in the same way and 300 μL of PBS (pH 7.4) was added to the silica gel and shaken vigorously, and left to equilibrate for 5 min at room temperature. This was the neutral release step. Both the peptide release ratios were quantified fluorimetric assay described above.

### Peptide digestive enzyme stability experiment

SGF (simulated gastric fluid) has been reported previously^41^. Sodium chloride (0.2 g) was added to a 100 mL flask and dissolved in 50 mL of pure water. Then, 0.7 mL of 10 M HCl was added to adjust the pH of the solution to 1.2. Next, 0.32 g of pepsin (161–24482; WAKO, Japan) was added and dissolved with gentle shaking and the volume was increased to 100 mL with pure water. Peptide degradation was performed using a thermostat (DTU-1B; TAITEC) at 37 °C. Firstly, 0.5 mM of DFELEDD peptide and 25 mg/mL silica gel solution were mixed. Secondly, 100 μL incubated SGF was added and the enzymatic reaction was conducted for 60 min. As a control experiment, 100 μL of phosphate buffer was added instead of SGF. After this, the sample was centrifuged and 200 μL of supernatant was added to 250 μL of 0.1 N NaOH to inactivate pepsin and the precipitate was added to 300 μL PBS to release the peptide. To quantify the peptide, 250 μL of solution was injected into the HPLC.

### Analysis of peptides by RP-HPLC

Chromatographic analysis was performed with a HPLC system (JASCO, Japan) equipped with pump (model PU 2086 Plus), UV detector (model MD 4017), and a C18 column (d = 20 mm, L = 250 mm; SHISEIDO, Japan). Solvent A contained 0.1% TFA in Milli Q water, and solvent B contained 0.1% TFA in acetonitrile. Separation of peptides was obtained using a linear gradient from 0% to 70% of solvent B for 35 min. The column was maintained at 30 °C, the flow rate was 5 mL/min, and the eluted peaks were detected by UV absorbance of 220 and 280 nm.

## Electronic supplementary material

Supplementary Information
