# Supplementary material for: Interaction between porous silica gel microcarriers and peptides for oral administration of functional peptides
Source: Sci Rep. 2018 Jul 20;8:10971. doi: 10.1038/s41598-018-29345-2 (PMC6054636; doi:10.1038/s41598-018-29345-2)
Supplement: Supplementary file 1 — Supplementary Information [file 41598_2018_29345_MOESM1_ESM.docx]

Supporting information

Interaction between porous silica gel microcarriers and peptides for oral administration of functional peptides

Kento Imai^1^, Kazunori Shimizu^1^, Mitsuhiro Kamimura^2^, Hiroyuki Honda^1,3^*

^1^ Department of Biomolecular Engineering, Graduate School of Engineering, Nagoya University, Nagoya 464-8603, Japan

^2^ FUJI SILYSIA CHEMICAL LTD., 1846, 2-Chome, Kozoji-Cho, Kasugai-Shi, Aichi,

487-0013, Japan

^3^ Innovative Research Center for Preventive Medical Engineering, Nagoya University, Nagoya 464-8601, Japan

**Table S1.** Physiochemical properties of tripeptides

**Table S2.** Physiochemical properties of pentapeptides

**Table S3.** Physiochemical properties of heptapeptides

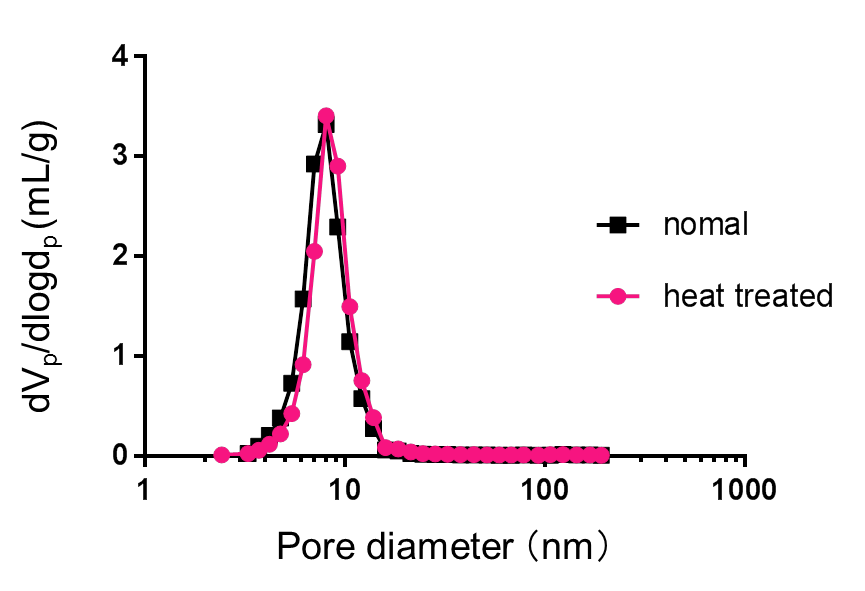


**Figure S1.** Pore size distributions determined by the BJH method.

**Figure S2.** Peptide adsorption ability by adding pentapeptide sequences. (A) A chart of the pentapeptide sequences classified by hydrophobicity versus isoelectric point (pI). Orange squares denote all tripeptides. Light blue diamonds denote representative peptides. (B) Peptide adsorption ability on heat-treated silica gel (pH 2.1). (C) Peptide adsorption ability on heat-treated silica gel (pH 7.4). (D) Score values for heat-treated silica gel. (E) Color map based on the results of (B). (F) Color map based on the results of (C). (G) Color map based on the results of (D).


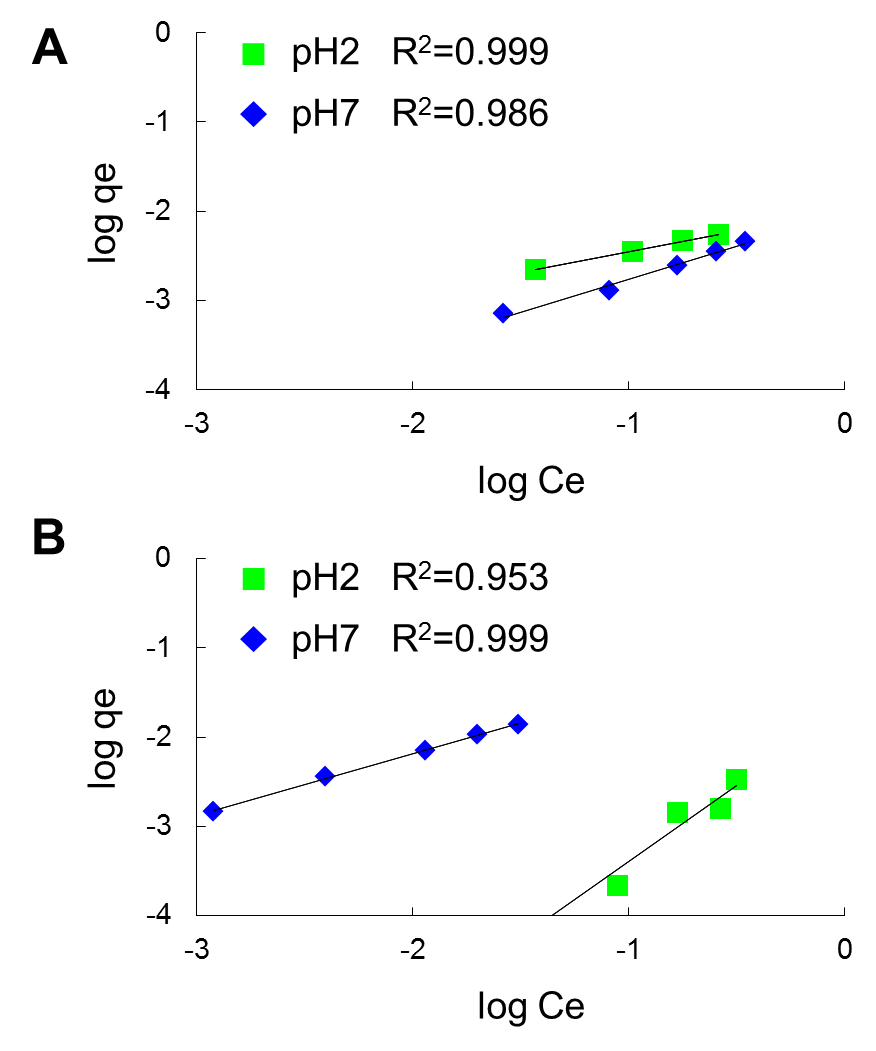


**Figure S3.** Freundlich fitting of isotherms for peptides. (A) VLDTDYK and (B) HNRNNRR.
